# Clinical characteristics and pathologic complete response (pCR) rate after neoadjuvant chemotherapy in postpartum women with breast cancer

**DOI:** 10.1007/s00432-023-05194-z

**Published:** 2023-08-09

**Authors:** He Dou, Siyuan Jia, Yuling Ba, Danli Luo, Pingyang Yu, Fucheng Li, Youyu Wang, Xingyan Chen, Min Xiao

**Affiliations:** Department of Breast Surgery, Harbin Medical University Cancer Hospital, No.150, Haping Road, Nangang District, Harbin, 150081 Heilongjiang People’s Republic of China

**Keywords:** Breast cancer, Neoadjuvant chemotherapy, Pathologic complete response, Pregnancy, Postpartum breast cancer

## Abstract

**Purpose:**

Breast cancer (BC) is currently the leading cause of death in women worldwide. Studies have confirmed that pregnancy is an independent factor affecting the survival of BC patients. BC found during pregnancy, lactation, or shortly after delivery is what we used to think of as pregnancy-associated breast cancer (PABC). The current expert definition of this concept is not uniform; however, there is growing evidence that postpartum breast cancer (PPBC) differs from other types of BC in terms of both biological features and prognosis, with a slightly different focus on diagnosis and treatment. With the increase of female reproductive age population and changes in fertility policies in China, patients with PPBC are receiving increasing attention. Here, we systematically analyzed the clinicopathological characteristics and chemotherapeutic response of patients with PPBC. We retrospectively analyzed the clinicopathological data, molecular subtypes, chemotherapy regimens, and pathological complete remission (pCR) rates of 1343 patients with non-metastatic BC at Harbin Medical University Cancer Hospital from January 1, 2012 to May 31, 2023. The categorical data were compared by chi-square test and Fisher exact test using logistic regression model. Predictor variables with *P* < 0.05 in the univariate analysis were included in the multivariate regression analysis to investigate the relationship between different age groups and pCR.

**Results:**

A total of 714 patients were eligible for analysis in this study, and 667 patients had a history of pregnancy, 40 (5.6%) of whom were PPBC patients. When diagnosed with BC, patients with PPBC were younger, more likely to undergo breast-conserving surgery (BCS), and more likely to achieve pCR *(P* < 0.05). In molecular typing, human epidermal growth factor receptor 2 (HER-2)-positive and triple-negative breast cancer (TNBC) were more frequent. In the entire cohort, HER-2 expression and delivery status were independent predictors of pCR rates in BC patients after neoadjuvant chemotherapy (NAC).

**Conclusion:**

Our findings suggest that postpartum status is an independent predictor of pCR attainment in BC patients. PPBC is more sensitive to chemotherapy than other patients.We need to pay more attention to this group and achieve individualized treatment, which will help us treat BC better and provide new targets and blueprints for our clinical therapy.

## Introduction

In recent years, the incidence of malignancies has been on the rise, and China has the highest annual incidence and mortality rate of malignancies in the world (Bray et al. 2018). After 1975, survival disparities among BC patients increased (Fan et al. 2014). By the end of 2020, the International Health Association reported that as many as 2.3 million people were diagnosed with BC, 126,000 more than in 2019. BC is increasing every year in China and has become the most common malignant disease in women (Johnson et al. 2013). In 2022, BC was listed as the fifth leading cause of cancer death in Chinese women. For BC, we advocate a three-stage approach of early detection, diagnosis, and treatment as much as possible. Currently, the treatment of BC tends to be diversified, and each treatment should be standardized and complete (Harbeck and Gnant 2017). Experts are also actively searching for treatments for BC.

Pregnancy is the growth and development of the embryo and fetus in the mother. The fertilization of a mature egg is the beginning of pregnancy, and the expulsion of the fetus and its appendages is the termination. Pregnancy is a very complex and highly coordinated physiological process (Gudnadottir et al. 2023). In recent years, the world economic landscape has gradually changed, and the pace of life has accelerated; modern people are under increasing employment and economic pressure, and raising the next generation requires more and more time and energy. As a result, childbirth is no longer the primary goal of today's young people (Jolly et al. 2000). According to the China Population Development Research Center, China’s one-child fertilitys rate declines from 0.7 to 0.5 between 2019 and 2022. In response to the aging population and declining fertility rate, China implemented a two-child policy on January 1, 2016, and a three-child policy on May 31, 2021, leading to a yearly increase in the age at which women become pregnant. According to the National Health Department and the Family Planning Commission, approximately 54 million women give birth at age 35 or older, which is extremely dangerous for women (Shan et al. 2018; Huang et al. 2023).

Older women are having children more frequently. In 2022, the average childbearing age in China was 29.1 years, well over 21.4 years in 1970, and experts believe that pregnancy is strongly associated with cancer incidence (Callihan et al. 2013). The relationship between BC and pregnancy is well known. BC is the most common pregnancy-related cancer in the world (Calsteren et al. 2010). BC is diagnosed during pregnancy in 0.2% of all BC cases, and the incidence has increased from 1 in 2000 in 1964 to 1 in 1000 (Czaplicki 2012). Pregnancy has a dual effect on BC, stopping the cancer’s progression and promoting metastasis (Proussaloglou et al. 2023).

Pregnancy is a function that is naturally endowed to humans. Most studies have shown that pregnancy has a strong protective effect on both humans and animals by slowing the development of BC, with each additional child resulting in a 5% reduction in the incidence of BC. Experts have found that menstruating women have a significantly lower risk of developing BC than women who have never given birth, because fetal cells in the mother’s tissues may help fight BC. Fetal cells may also provide the mother with immune surveillance for cancer cells, keeping the mother's immune system more alert. Meier found that the earlier a woman's pregnancy is, the more protective mechanisms are evident. The protective effect of the first pregnancy after age 30 was much later, and essentially no protective effect occurred in women who delivered at age 35 (Meier-Abt and Bentires-Alj 2014). Ploquin found that breast cancer in pregnancy (BCP) had the same survival outcome as women who did not deliver if patients were treated appropriately (Fortner et al. 2019; Ploquin et al. 2018). Sun stratified patients by age, and he found that pregnancy had little impact on the survival prognosis of BC patients, which was primarily driven by tumor biology (Sun and Lee 2020). However, some experts have different opinions. Schedin believes all patients with transitional delivery have a 10–30% increased risk of BC for at least ten years after delivery, the older the first pregnancy (Schedin 2006). Muñoz found that the protective effect of pregnancy depends on the woman's age at the time of her first pregnancy, which is of most significant benefit to young mothers. BC diagnosed 5–10 years after delivery had a higher risk of metastasis, and the crossover effect eliminated this risk over time (Muñoz-Montaño et al. 2021). When an adult woman gives birth, the significant change is postpartum breast degeneration, a process of breast degeneration in which the epithelial and lactational state of the breast degenerates and returns to its pre-pregnancy state after a period of tissue remodeling (Lyons et al. 2009). About 80–90% of normal breast epithelial tissue is removed through programmed death (Walker et al. 1989). Tumor cells can grow and progress in this wound-healing environment, a vitai risk window for BC development (Lund et al. 1996). In addition, breast degeneration is primarily characterized by increased collagen deposition, increased immune cell infiltration, increased cytokine influx, and increased bioactive fragmentation of protein hydrolysis targets. This microenvironmental change also drives its tumorigenesis (Clarkson et al. 2004; Stein et al. 2004). It is thus clear that pregnancy is a double-edged sword and cannot be considered a single risk or protective factor.

PPBC is a global health threat that affects approximately 150,000 to 350,000 young mothers each year, who face a higher risk of cancer and death and a much higher risk of dying. There is no clear definition of PPBC, and BC is generally considered to be diagnosed within five years of delivery (Callihan et al. 2013; Harvell et al. 2013). Previous studies on PPBC were mostly from Western countries, with only a few from Asia. Azim conducted a meta-analysis of 30 studies and found that BCP and PPBC patients had a worse prognosis than other BC patients (Azim et al. 2012). Rodriguez compared 797 PPBC cases with 4177 non-PPBC control cases. PPBC exhibited more advanced disease, larger tumors, and a higher percentage of hormone receptor (HR) negative tumors (Rodriguez et al. 2008). The risk of death in PPBC is also higher when controlling for age, race, and HR. Various theories have been proposed regarding why PPBC patients have a low survival rate. Tretli found a significant increase in estrogen levels during or shortly after pregnancy, and estrogen isa carcinogen in BC (Tretli et al. 1988). Asselin found that mammary stem cells during pregnancy respond strongly to steroid signals and that their temporary increase may be responsible for the more aggressive nature of PPBC (Asselin-Labat et al. 2010). And the combined effects of immunosuppression and increased angiogenesis will also play a role during pregnancy.

PPBC as a distinct BC subtype remains highly controversial, probably due to the lack of reliable biomarkers to diagnose PPBC, and further studies on the tumor biology of PPBC are needed to find better therapeutic interventions to prevent disease recurrence and progression in this population. Therefore, by analyzing the clinicopathological characteristics of PPBC patients and the pCR rate after NAC compared to other BC patients, we can find out whether pregnancy affects the response of NAC patients so that we can improve the understanding of the “postpartum effect” and choose a better treatment option.

## Material and methods

### Study sample

We conducted a retrospective cohort study that collected patients treated at Harbin Medical University Cancer Hospital from January 1, 2012, to May 31, 2023, who underwent physical examination, imaging (ultrasound, x-ray), and hollow needle biopsy of breast masses confirmed as BC, after communicating with the patients and their families and agreeing to NAC. Beforetreatment, the patients and their families were explained individual clinical and pathological information for clinical studies. The patient signed an informed consent regarding the medical history data and secondary use of the biospecimen. Patients underwent chemotherapy according to standard guidelines with a complete cycle of chemotherapy; surgical treatment was performed after completion of NAC, with mastectomy or breast-conserving surgery (BCS) depending on the patient's condition and wishes, and all patients underwent sentinel lymph node biopsy (SLNB) and axillary lymph node dissection (ALND) if lymph nodes had metastasized.

### Inclusion and exclusion criteria

**Fig. 1:**
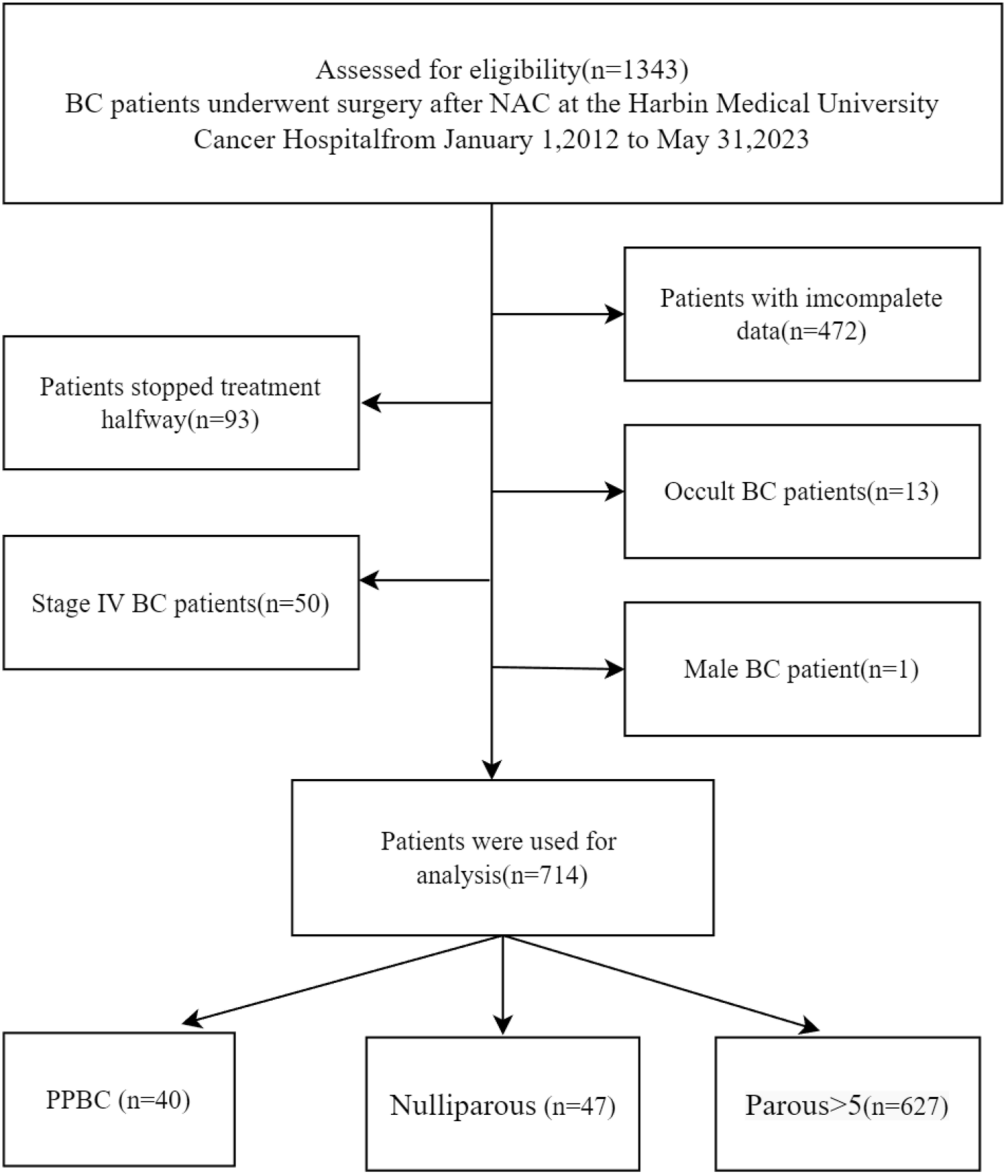
Grouping flow chart of 714 breast cancer patients collected

A total of 1343 patients were obtained for analysis, and detailed inclusion criteria included (1) female patients; (2) pathologically confirmed BC prior to chemotherapy; (3) all patients received NAC and completed treatment; (4) complete clinical and pathological data; (5) T1-T3 tumors as defined by the AJCC TNM staging system; and (6) patients underwent pathological IHC testing at the beginning and end of NAC. Exclusion criteria included (1) patients with incomplete data, (2) patients with multiple tumors, (3) patients with insufficient age at diagnosis and life status, (4) patients with occult BC, (5) male patients, and (6) patients with interrupted treatment or treatment at other hospitals. Finally, 714 patients who met this index were selected for analysis. This flow chart is shown in Fig. 1. The baseline characteristics of this cohort in this study are shown in Table 1.

**Table 1:** Descriptive characteristics and treatments of BC patients by parity status

Patients characteristic	Parity status			*P*
	Nulliparous	PPBC	Parous > 5	
	*N*(%)	*N*(%)	*N*(%)	
Total	47 (6.6)	40 (5.6)	627 (87.8)	
Age at diagnosis				
≤ 40	22 (46.8)	33 (82.5)	61 (9.7)	** < 0.001**
> 40	25 (53.8)	7 (17.5)	566 (90.3)	
Surgical methods				
BCS	6 (12.8)	7 (17.5)	21 (3.3)	** < 0.001**
M	41 (87.2)	33 (82.5)	606 (96.7)	
Menstruatio				
Yes	13 (27.7)	1 (2.5)	333 (53.1)	** < 0.001**
No	34 (72.3)	39 (97.5)	294 (46.9)	
BMI				
≤ 18.5	1 (2.1)	2 (5.0)	13 (2.2)	0.564
18.5–24	25 (53.2)	20 (50.0)	263 (41.8)	
24–30	19 (40.4)	16 (40.0)	311 (49.6)	
≥ 30	2 (4.3)	2 (5.0)	40 (6.4)	
Clinical T stage				
1	6 (12.8)	5 (12.5)	75 (11.9)	0.245
2	28 (59.6)	26 (65.0)	452 (72.0)	
3	13 (27.6)	9 (22.5)	100 (16.1)	
Clinical N stage				
0	6 (12.8)	7 (17.5)	83 (13.2)	0.984
1	25 (53.2)	20 (50.0)	332 (52.9)	
2	6 (12.8)	6 (15.0)	78 (12.4)	
3	10 (21.2)	7 (17.5)	134 (21.5)	
Lymphatic infiltration				
Yes	31 (66.0)	32 (80.0)	470 (75.0)	0.421
No	16 (34.0)	8 (20.0)	157 (25.0)	
ER				
Positive	31 (66.0)	17 (42.5)	349 (55.6)	0.090
Negative	16 (34.0)	23 (57.5)	278 (44.6)	
PR				
Positive	23 (48.9)	16 (40.0)	273 (43.6)	0.686
Negative	24 (51.1)	24 (60.0)	354 (56.4)	
HER-2				
Positive	16 (34.0)	18 (45.0)	232 (36.9)	0.535
Negative	31 (66.0)	22 (55.0)	395 (63.1)	
KI67				
≤ 15	12 (25.5)	13 (32.5)	216 (34.4)	0.453
> 15	35 (74.5)	27 (67.5)	411 (65.6)	
P53				
0	30 (63.8)	21 (52.5)	305 (48.6)	0.583
1	8 (17.0)	11 (37.5)	160 (25.5)	
2	4 (8.6)	4 (10.0)	76 (12.1)	
3	5 (10.6)	4 (10.0)	86 (13.8)	
Histological grade				
0–1	16 (34.0)	17 (42.5)	218 (34.7)	0.789
2	27 (57.4)	19 (47.5)	332 (53.0)	
3	4 (8.6)	4 (10.0)	77 (12.3)	
Stage				
I	0 (0)	1 (2.5)	11 (1.8)	0.828
II	25 (53.2)	22 (55.0)	359 (57.3)	
III	22 (46.8)	17 (42.5)	257 (40.9)	
Molecular subtype				
HR+/HER-2 +	9 (19.1)	6 (15.0)	71 (11.3)	0.140
HR +/HER-2 –	22 (46.8)	11 (27.5)	283 (45.1)	
HR –/HER-2 +	7 (14.9)	12 (30.0)	162 (25.8)	
TNBC	9 (19.2)	11 (27.5)	111 (17.8)	
Histological type				
IDC	31 (66.0)	25 (62.5)	442 (70.5)	0.280
ILC	5 (10.6)	3 (7.5)	69 (11.0)	
DCIS	3 (6.4)	4 (10.0)	19 (3.0)	
Others	8 (17.0)	8 (20.0)	97 (15.5)	
Chemotherapy regimen				
Anthracycline-based	42 (89.4)	34 (85.0)	568 (90.6)	0.504
Other therapy	5 (10.6)	6 (15.0)	59 (9.4)	

Bold values indicate that they are statistically significant at *P* ≤ 0.05

This research complies with the World Medical Association Declaration of Helsinki 1964 and subsequently amended versions. All of the patients signed an informed consent form before the treatment.

### Clinical and pathological variables

Study variables included patient age, delivery history, surgical procedure, menopausal status, body mass index (BMI) value, lymphatic infiltration status, ER status, PR status, HER-2 status, KI67 expression, P53 expression, T-stage, N-stage, clinical stage, molecular subtype, chemotherapeutic drug type, pathological type, histological grade, and pCR status. Patient information and treatment details were recorded from the time of diagnosis. Patients were divided into three groups according to delivery: as follows: Nulliparous, PPBC, and Parous > 5 years. Patients with PPBC were diagnosed with BC within 5 years of pregnancy. Age was divided into two groups using 40 years as a cut-off. Surgical procedures were divided into mastectomy and BCS. Natural menopause was defined as patients with over 12 months of future menstruation or over 60 years of age. BMI values were stratified according to international health standards: thin, BMI < 18.5; normal, 18.5 ≤ BMI < 24; overweight, 24 ≤ BMI < 30, and obese BMI ≥ 30. IHC was used to detect the HR status of patients, and positive ER and PR expression was defined as 1% nuclear staining of tumor cells. HER-2 was positive when IHC stained 3 + and negative when IHC stained 0 or 1 + HER-2. When IHC stained 2 + , its status was detected by fluorescence in situ hybridization (FISH), and HER-2 was considered negative when Fish was negative. Otherwise, it is a positive one. KI67 refers to an anti-germin monoclonal antibody, a proliferating cell nuclear antigen associated with the tumor cell cycle, interpreted as the percentage of tumor cell nuclei between 400 and 500 cells. KI67 positive nuclear ≥ 15% was defined as high expression, < 15% as low expression. Clinical and imaging staging was performed in all patients. T-stage staging was determined by palpation and ancillary examinations. N-stage was defined as abnormal axillary lymph nodes or lymph nodes detected by ultrasound. Metastatic disease was assessed by imaging. We classify cancer molecular subtypes into four types: HR(+)/HER-2(+), HR(+)/HER-2(−), HR(−)/HER-2(+), and TNBC. Pathologists observe tumor sections and analyze pathological types, like IDC, ILC, etc. According to the pathological assessment after NAC of the Chinese Society of Clinical Oncology, the Miller & Payne system is currently used to evaluate primary lesions. This system mainly compares pre- and post-treatment surgical specimens.

To assess the abundance of residual infiltrating tumor cells after NAC, specific interpretation criteria are divided into the following five levels: Grade 1 (G1): no change in infiltrating cancer cells or only a single cancer cell is changed, and there is no decrease in the number of tumor cells; Grade 2 (G2): mild decrease in infiltrating cancer cells, but the total number is still high, and the decrease does not exceed 30%; Grade 3 (G3): decrease in infiltrating cancer cells by 30–90%; Grade 4 (G4): decrease in infiltrating cancer cells by more than 90% and only small clusters or a few scattered single cancer cells remain; Grade 5 (G5): no infiltrating cancer cells at the original tumor bed site, but ductal carcinoma in situ (DCIS) may be present. G5 is used here as the study endpoint for this cohort.

### Pathological detection and immunohistochemistry

IHC is a globally accepted method for detecting ER and PR in BC. Pathologists or pathology assistants cut tumor and benign breast tissue samples. They were placed in the same container during the initial evaluation, thus ensuring that typical breast structures were used as subsequent controls. All study results were reviewed and confirmed by both pathologists. Pathological sections were 4 μm thick and specimens were embedded in paraffin and incubated with primary antibody, secondary antibody, and streptavidin in sequence.

### Statistical analysis

The data presented in this paper were analyzed using SPSS software version 26.0 (IBM Corporation, New York, USA) and the data presented in this paper were stratified by pregnancy status. Categorical data were expressed as counts and percentages and continued to be compared and analyzed using chi-square tests and univariate logistic regression analysis for correlation of clinical case parameters with pCR rates within each subgroup. Statistically significant variables for univariate analysis were included in multivariate analysis. To determine which variables were independent predictors of pCR, *P* < 0.05 was considered statistically significant. The nomogram was established based on clinicopathologic factors of BC patients. In addition, we analyzed the overall performance of the nomogram by plotting the receiver operating characteristic (ROC) curves and then calculating the AUC of the ROC curves with the aim of analyzing the overall performance of the nomogram, with the AUC exceeding 0.7 considered that the nomogram provided a reasonable estimation, and the above statistical analyses were performed by R4.1.0 software, including the car, rms, pROC, and rmda package.

## Results

### Characteristics of study sample

**Fig. 2:**
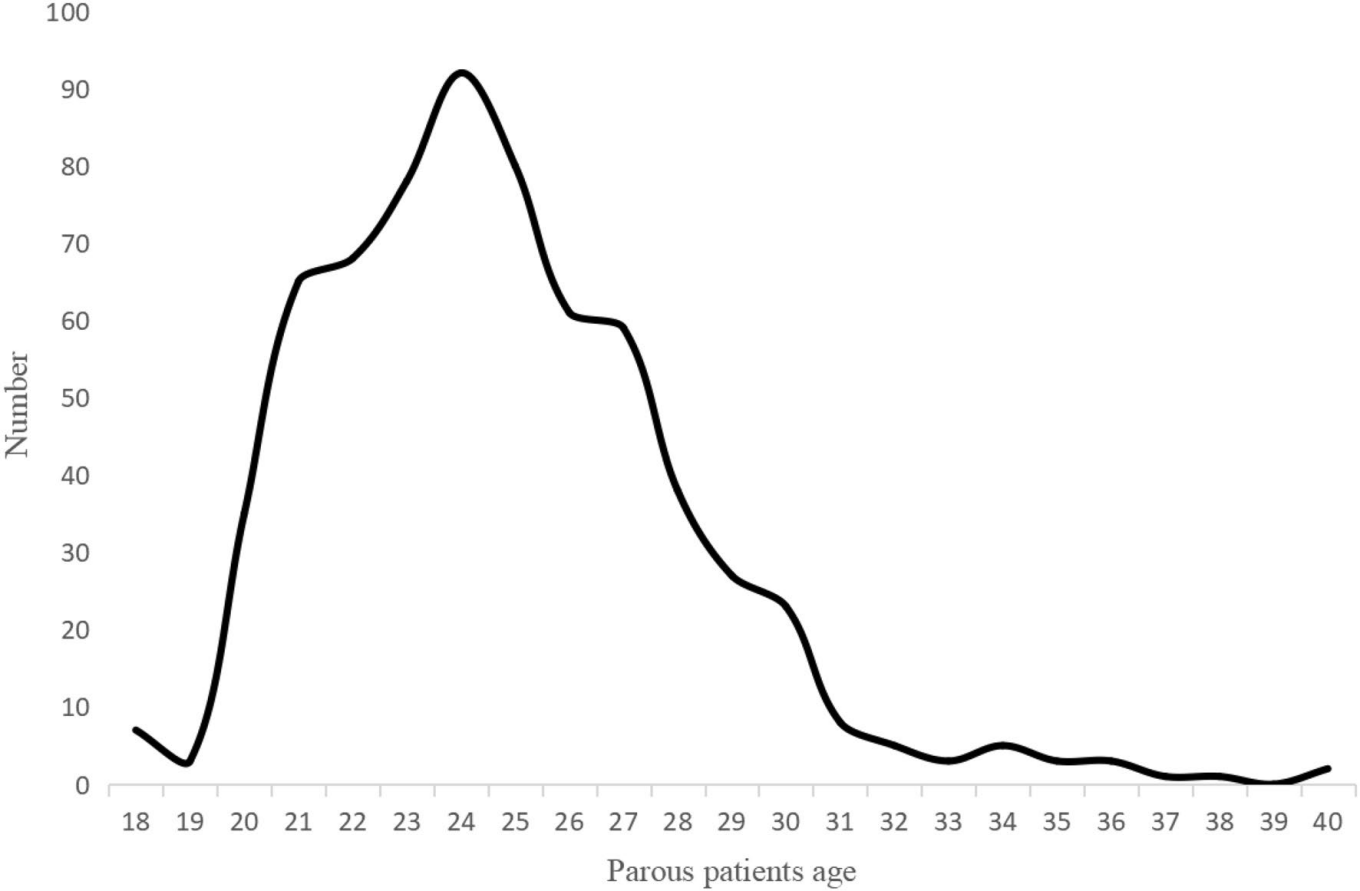
Age distribution of postpartum patients

From January 1, 2012, to May 31, 2023, 1343 patients diagnosed with BC were studied at Harbin Medical University Cancer Hospital, 629 patients were excluded (472 patients without complete information, 93 patients discontinued or transferred, 13 patients diagnosed with occult BC, 1 male patient, 50 stage IV BC), and a total of 714 were included in the study. A total of 667 BC patients had a history of pregnancy, 40 patients with PPBC, 627 BC patients with Parous > 5 years, and 40 unproductive patients. The clinicopathological characteristics and treatment are shown in Table 1. The age distribution of the patients who delivered is shown in Fig. 2. The age range was 21–71, with a median age of 48. More women gave birth at the age of 24 years. Different gestational status was strongly associated with age at diagnosis, menopausal status, and surgical procedure (*P* < 0.05). In this cohort, PPBC patients were younger than other types of patients. 33 patients (82.5%) were diagnosed with BC before the age of 40 years, so most patients were in premenopausal status, and menstrual status was strongly correlated with age. The probability of positive HER-2 expression was higher in PPBC patients (45%) than in controls, and 11 patients (27.5%) presented with TNBC, but it was not statistically significant. Among all known molecular subtypes of BC, TNBC had the worst prognosis. Most clinicopathological features did not differ significantly between groups regarding parity (*P* > 0.05). In the entire cohort, younger patients were more likely to undergo BCS, but the overall breast-conservation rate was not high, at 4.8%. This may be related to the prevailing treatment setting and patient mindset. 90.2% of patients received anthracycline chemotherapy, the majority were IDC (69.3%), and the most common molecular subtype was HR(+)/HER-2(–) (44.3%).

### Relationship between clinical factors and pCR in the maternal group

Among patients who delivered, 111 (16.6%) achieved pCR and 556 (83.4%) did not. The univariate analysis determined the factors affecting the pCR rate after NAC. Age, T stage, N stage, ER expression, PR expression, HER-2 expression, KI67 expression, histological grade, molecular subtype, clinical stage, type of pathology, and time of delivery were all strongly associated with pCR rate (*P* < 0.05) (Table 2). However, there was no significant correlation between chemotherapy regimen, surgical mode, menopausal status, BMI, P53 expression, lymphatic infiltration, and pCR (*P* > 0.05). Patients with younger age, lower T- and N-stage, ER-negative, PR-negative, HER2-positive, high KI67 expression, and lower BC histological grade were more likely to achieve pCR.

**Table 2:** Univariate analysis between clinical characteristics and pCR

Patients characteristic	Postpartum patients				*χ*2	*P*
	pCR (*n* = 111)		NpCR (*n* = 556)			
	*N*	%	*N*	%		
Age						
≤ 40	25	22.5	69	12.4	**7.815**	**0.005**
> 40	86	77.5	487	87.6		
Surgical methods						
BCS	4	3.6	24	4.3	0.118	0.732
M	107	96.4	532	95.7		
Menstruatio						
Yes	57	51.3	277	49.8	0.087	0.768
No	54	48.7	279	50.2		
BMI						
≤ 18.5	1	0.9	14	2.5	3.566	0.312
18.5–24	53	47.7	230	41.3		
24–30	48	43.2	279	50.2		
≥ 30	9	8.2	33	6.0		
Clinical T stage						
1	22	19.8	58	10.4	**8.736**	**0.013**
2	76	68.5	402	72.3		
3	13	11.7	96	17.3		
Clinical N stage						
0	24	21.6	66	11.9	**10.216**	**0.017**
1	60	54.1	292	52.5		
2	9	8.1	75	13.5		
3	18	16.2	123	22.1		
Lymphatic infiltration						
Yes	85	76.6	417	75.0	0.124	0.725
No	26	23.4	139	25.0		
ER						
Positive	34	30.6	332	59.7	**31.602**	** < 0.001**
Negative	77	69.4	224	40.3		
PR						
Positive	23	20.7	266	47.8	**27.717**	** < 0.001**
Negative	88	79.3	290	52.2		
HER-2						
Positive	61	55.0	189	34.0	**17.351**	** < 0.001**
Negative	50	45.0	367	66.0		
KI67						
≤ 15	27	24.3	202	36.3	**5.916**	**0.015**
> 15	84	75.7	354	63.7		
P53						
0	46	41.4	280	50.4	5.070	0.167
1	28	25.2	143	25.7		
2	16	14.4	64	11.5		
3	21	19.0	69	12.4		
Histological type						
IDC	7	6.3	460	82.7	**466.983**	** < 0.001**
ILC	1	0.9	71	12.8		
DCIS	19	17.1	2	0.3		
Others	84	75.7	23	4.2		
Chemotherapy regimen						
Anthracycline-based	98	88.3	504	90.6	0.586	0.444
Other therapy	13	11.7	52	9.4		
Histological grade						
0–1	108	97.3	126	22.6	**226.348**	** < 0.001**
2	3	2.7	349	62.8		
3	0	0	81	14.6		
Stage						
I	3	2.7	9	1.6	**11.253**	**0.004**
II	78	70.3	302	54.3		
III	30	27.0	245	44.1		
Molecular subtype						
HR +/HER-2 +	14	12.6	63	11.3	**37.211**	** < 0.001**
HR +/HER-2 −	21	18.9	273	49.1		
HR −/HER-2 +	47	42.3	127	22.8		
TNBC	29	26.2	93	16.8		
Parity status						
PPBC	15	13.5	25	4.5	**13.345**	** < 0.001**
Parous > 5	96	86.5	531	95.5		

Bold values indicate that they are statistically significant at *P* ≤ 0.05

Factors that were statistically significant in the univariate analysis were entered into the multifactorial analysis (both of which were excluded due to differences in histological grading and type of pathology that could affect the results). Logistic regression analysis showed that patients with PPBC were more likely to achieve PCR compared with those who had been in labor for more than 5 years (OR = 2.526, CI 95% 1.026–6.219, *P* = 0.040). HER-2 positive patients were more likely to achieve pCR than negative ones (OR = 1.804, CI 95% 1.130–2.879, *P* = 0.013). These two factors were statistically significant. Delivery status and HER-2 expression were strongly associated with the performance of PCR in BC patients (*P* < 0.05) and were independent predictors of achieving postoperative PCR (Table 3).

**Table 3:** Multivariate analysis between clinical characteristics and pCR

Patients characteristic	B	S.E	Wals	OR	CI (95%)	*P*
Age						
> 40	Ref					
≤ 40	0.411	0.357	1.325	1.509	0.749–3.040	0.250
Clinical T stage						
3	Ref					
1	0.904	0.495	3.335	2.468	0.936–6.509	0.067
2	0.139	0.416	0.111	0.870	0.385–1.968	0.739
ER						
Positive	Ref					
Negative	0.579	0.338	2.929	1.783	0.919–3.456	0.087
PR						
Positive	Ref					
Negative	0.649	0.375	2.989	1.914	0.917–3.995	0.084
HER-2						
Negative	Ref					
Positive	0.590	0.239	6.120	1.804	1.130–2.879	**0.013**
Parity status						
Parous > 5	Ref					
PPBC	0.927	0.460	4.063	2.526	1.026–6.219	**0.044**
KI67						
≤ 15	Ref					
> 15	0.449	0.261	2.952	1.567	0.939–2.616	0.086
Clinical N stage						
3	Ref					
0	0.026	0.705	0.001	1.027	0.258–4.084	0.970
1	0.705	0.623	1.280	0.494	0.146–1.676	0.258
2	0.206	0.462	0.199	0.814	0.329–2.013	0.655
Stage						
III	Ref					
I	0.157	1.081	0.021	1.170	0.141–9.747	0.884
II	1.364	0.629	4.693	3.910	1.139–13.426	**0.030**

Bold values indicate that they are statistically significant at *P* ≤ 0.05

### Predictive analysis of patients obtaining pCR or NpCR

**Fig. 3:**
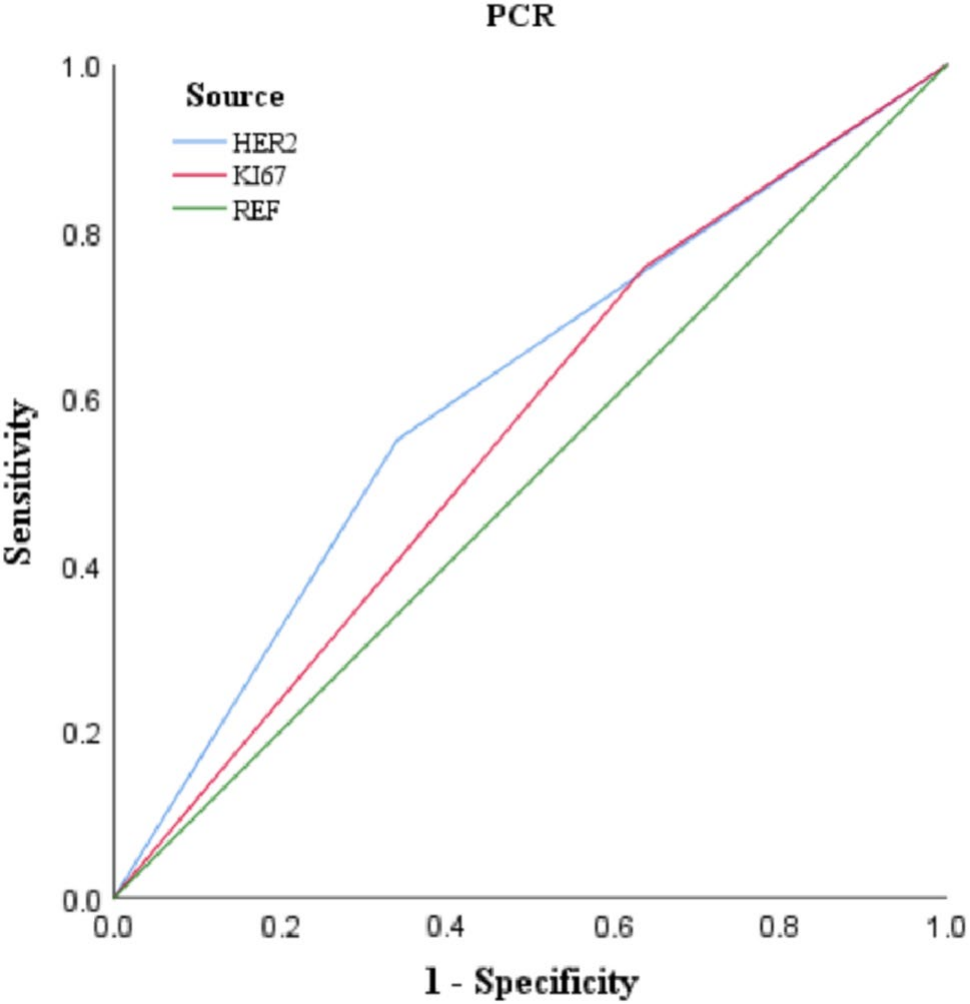
The ROC curves for HER-2 and KI67

**Fig. 4:**
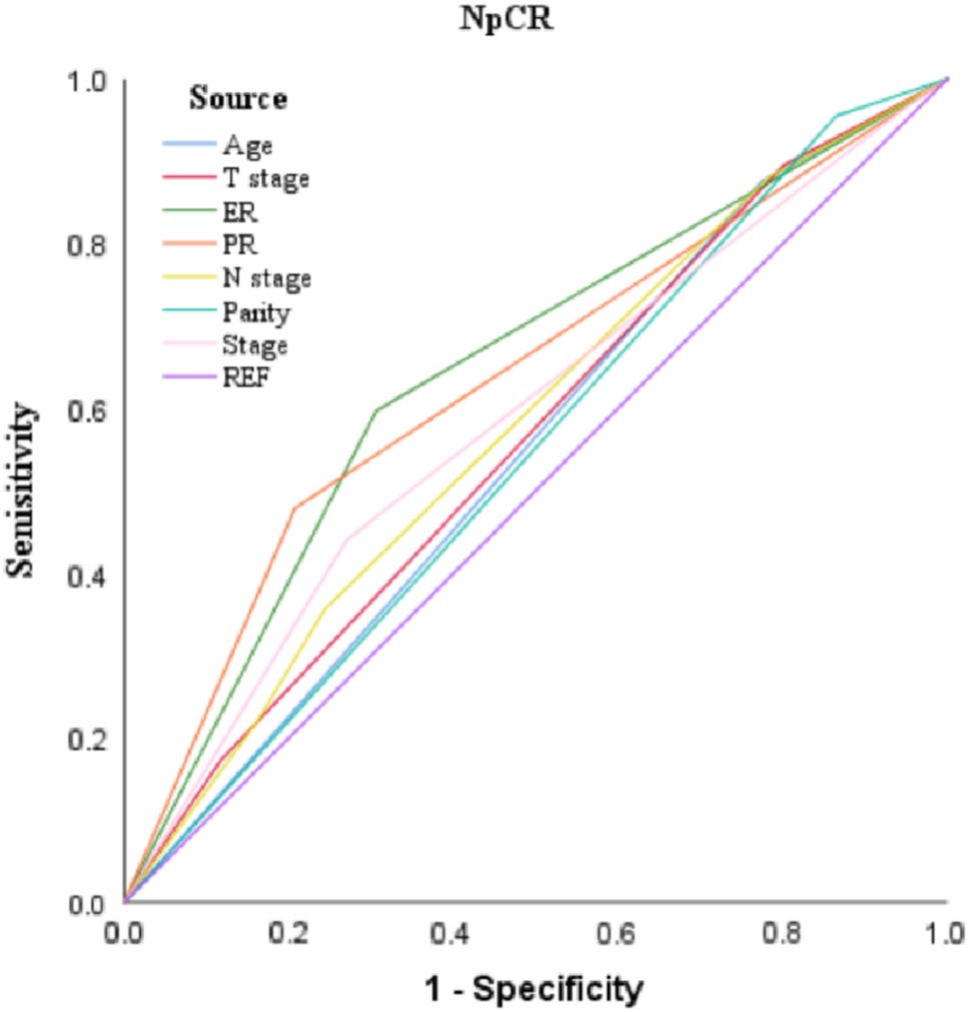
The ROC curves for Age, T stage, ER, PR, N stage, Parity and Stage

We used the ROC to predict the probability of obtaining pCR in BC patients (Figs. 3, 4). The results revealed that the probability of obtaining pCR was 56.0% for KI67 > 15 patients and 60.5% for HER- 2-positive patients (Table 4), with statistically significant differences (*P* < 0.05). In addition, this study also found that when clinical T or N staging was high, and ER or PR expression was positive, patients were less likely to achieve pCR, with probabilities of 56.4%, 57.9%, 64.5%, and 63.6%, respectively, with statistically significant differences (*P* < 0.05) (Table 5). BC patients were less likely to achieve pCR when women had been in labor for more than 5 years, but the difference was not statistically significant (*P* > 0.05).

**Table 4:** Predictive analysis of KI67 and HER-2 expression affecting pCR

Patients characteristic	AUC	S.E	CI (95%)	*P*
HER-2				
Negative	Ref			
Positive	0.605	0.030	0.546–0.663	** < 0.001**
KI67				
≤ 15	Ref			
> 15	0.560	0.029	0.503–0.617	**0.046**

Bold values indicate that they are statistically significant at *P* ≤ 0.05

**Table 5:** Predictive analysis of stage, age and PR expression affecting NpCR

Patients characteristic	AUC	S.E	CI (95%)	*P*
Age				
≤ 40	Ref			
> 40	0.551	0.031	0.489–0.612	0.092
Clinical T stage				
1 + 2	Ref			
3	0.564	0.030	0.504–0.623	**0.034**
ER				
Negative	Ref			
Positive	0.645	0.028	0590–0.701	** < 0.001**
PR				
Negative	Ref			
Positive	0.636	0.027	0.583–0.689	** < 0.001**
Clinical N stage				
0	Ref			
1, 2, 3	0.579	0.030	0.520–0.638	**0.008**
Parity status				
PPBC	Ref			
Parous > 5	0.545	0.031	0.484–0.607	0.133
Stage				
I	Ref			
TT, III	0.587	0.029	0.531–0.0.643	**0.004**

Bold values indicate that they are statistically significant at *P* ≤ 0.05

### Construction of nomogram-based prediction of pCR in patients with BC after receiving NAC

**Fig. 5:**
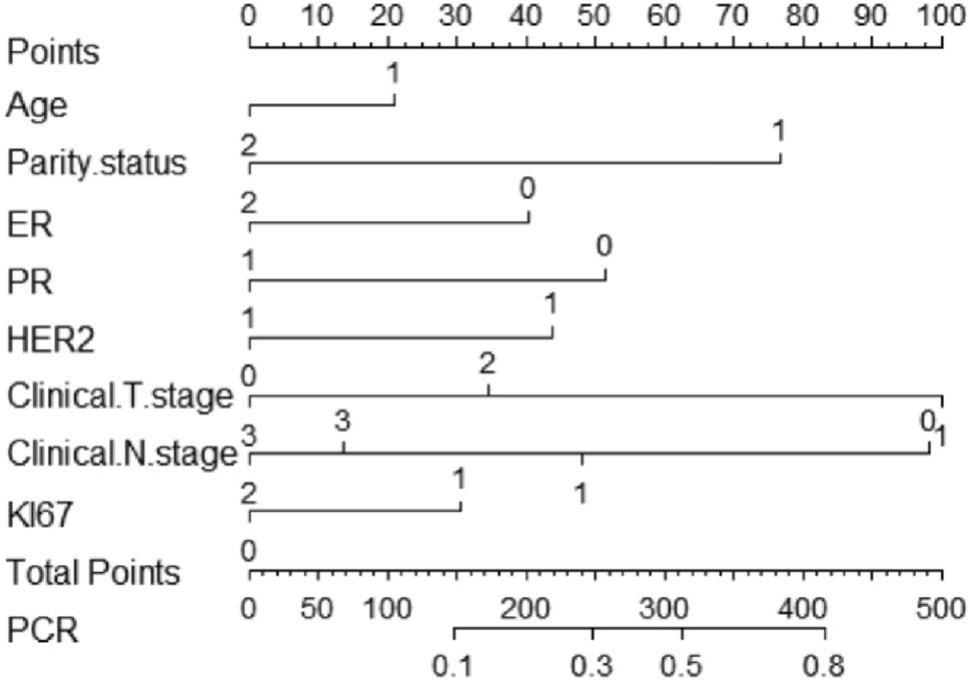
Nomogram to predict the probability of reaching pCR in BC patients

**Fig. 6:**
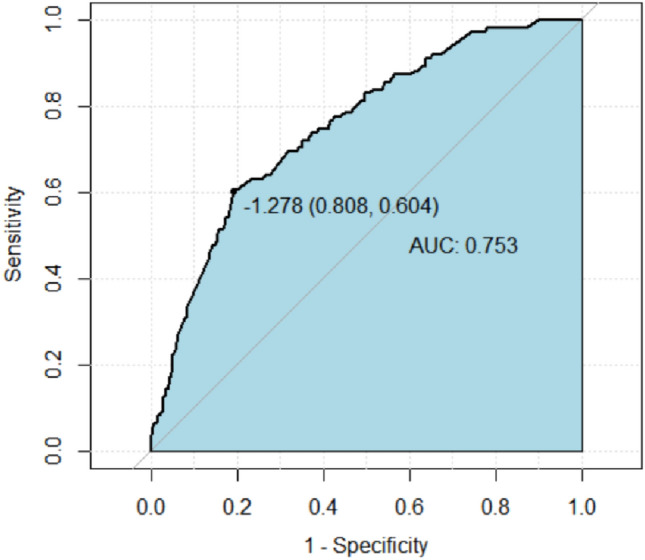
ROC curve and the area under the curves of ROC curves in the training cohor

A nomogram based on clinicopathologic characteristics of BC patients was designed to predict the probability of reaching pCR when BC patients are NAC. Clinical T and N staging had the greatest impact on pCR, and pregnancy status also had a large impact, with PPBC patients more likely to reach pCR (Fig. 5). To assess the predictive ability of the a nomogram graph-based prediction model to achieve pCR in BC patients, we used the ROC curve. the AUC was 0.753 (95% CI: 0.604–0.808), indicating that the prediction model had good judgment (Fig. 6).

### Correlation between clinical factors and pCR in the PPBC and Parous > 5 group

**Fig. 7:**
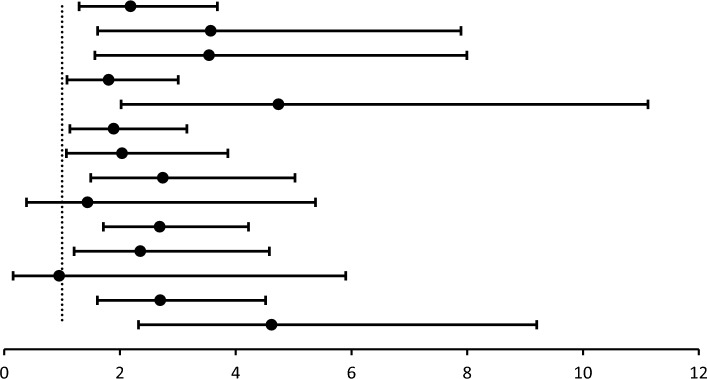
Image description of RR values in PPBC patients (in the order of Table 8)

The present study analyzed the pCR rates for clinicopathological features in the different delivery groups (Table 6). In most cases, patients with PPBC were more likely to achieve pCR than those who had been in labor for more than 5 years, and this relationship was significant. We further analyzed the interaction between time to delivery and clinicopathological features of BC by general linear model (Table 7). patients with PPBC were associated with age, lymphatic infiltration, ER expression, PR expression, HER-2 expression, KI67 expression, p53 expression, and clinical stage, and RR values were calculated between subgroups (*P* < 0.001) (Fig. 7). patients with PPBC were more likely to reach pCR, a phenomenon that was only insignificant when BC patients were older.

**Table 6:** Relationship with the clinical characteristics and pCR according to parous age group

Patients characteristic	Postpartum patients				*χ*^2^	*P*
	pCR (*n* = 111)		NpCR (*n* = 556)			
	*N*	%	*N*	%		
Age at diagnosis						
≤ 40						
PPBC	14	12.6	19	3.4	**6.526**	**0.011**
Parous > 5	11	9.9	50	9.0		
> 40						
PPBC	1	0.9	6	1.1	0.003	0.957
Parous > 5	85	76.6	481	86.5		
Menstruatio						
Yes						
PPBC	15	13.5	1	0.1	**54.753**	** < 0.001**
Parous > 5	57	51.3	276	49.6		
No						
PPBC	0	0	24	4.3	3.629	0.057
Parous > 5	39	35.2	255	46.0		
Surgical methods						
BCS						
PPBC	1	0.9	5	0.8	0.241	0.623
Parous > 5	2	1.8	19	3.4		
M						
PPBC	14	12.6	20	3.6	**15.117**	** < 0.001**
Parous > 5	94	84.7	512	92.2		
ER						
Positive						
PPBC	5	4.5	12	2.2	**8.566**	**0.003**
Parous > 5	29	26.1	320	57.6		
Negative						
PPBC	10	9.0	13	2.3	**4.190**	**0.041**
Parous > 5	67	60.4	211	37.9		
PR						
Positive						
PPBC	5	4.5	11	2.0	**12.544**	** < 0.001**
Parous > 5	18	16.2	255	45.9		
Negative						
PPBC	10	9.0	14	2.5	**4.851**	**0.028**
Parous > 5	78	70.3	276	49.6		
HER-2						
Positive						
PPBC	7	6.3	11	2.0	**3.989**	**0.046**
Parous > 5	42	37.8	178	32.0		
Negative						
PPBC	8	7.2	14	2.5	**9.005**	**0.003**
Parous > 5	54	48.7	353	63.5		
Lymphatic infiltration						
Yes						
PPBC	11	9.9	21	3.8	**7.393**	**0.007**
Parous > 5	74	66.7	396	71.2		
No						
PPBC	4	3.6	4	0.7	**7.426**	**0.006**
Parous > 5	22	19.8	135	24.3		
Clinical T stage						
1						
PPBC	1	0.9	4	0.7	0.150	0.698
Parous > 5	21	18.9	54	9.7		
2						
PPBC	9	8.1	17	3.1	**7.203**	**0.007**
Parous > 5	67	60.3	385	69.2		
3						
PPBC	5	4.5	4	0.7	**17.777**	** < 0.001**
Parous > 5	8	7.3	92	16.6		
Clinical N stage						
0						
PPBC	4	3.6	3	0.5	3.605	0.058
Parous > 5	20	18.0	63	11.3		
1						
PPBC	6	5.4	14	2.5	2.517	0.113
Parous > 5	54	48.7	278	50.0		
2						
PPBC	3	2.7	3	0.5	**10.425**	**0.001**
Parous > 5	6	5.4	72	12.9		
3						
PPBC	2	1.8	5	0.9	1.652	0.199
Parous > 5	16	14.4	118	21.4		
KI67						
≤ 15						
PPBC	2	1.8	10	2.0	0.282	0.595
Parous > 5	25	22.5	191	34.3		
> 15						
PPBC	13	11.7	15	2.7	**14.399**	** < 0.001**
Parous > 5	71	64.0	340	61.0		
P53						
0						
PPBC	5	4.5	1	0.1	**22.806**	** < 0.001**
Parous > 5	41	36.9	264	47.5		
1						
PPBC	5	4.5	2	0.4	**15.643**	** < 0.001**
Parous > 5	23	20.7	137	24.6		
2						
PPBC	3	2.7	16	2.9	0.019	0.891
Parous > 5	13	11.7	63	11.3		
3						
PPBC	2	1.8	6	1.1	0.036	0.850
Parous > 5	19	17.2	67	12.1		
Chemotherapy regimen						
Anthracycline-based						
PPBC	13	11.7	21	3.8	**12.746**	** < 0.001**
Parous > 5	85	76.6	483	86.8		
Other therapy						
PPBC	2	1.8	4	0.7	0.734	0.391
Parous > 5	11	9.9	48	8.7		
Stage						
I + II						
PPBC	8	7.2	15	2.7	2.971	0.085
Parous > 5	73	65.7	296	53.2		
III						
PPBC	7	6.3	10	1.8	**17.080**	** < 0.001**
Parous > 5	23	20.8	235	42.3		
Molecular subtype						
HR + /HER-2 +						
PPBC	1	0.9	5	0.9	0.010	0.920
Parous > 5	13	11.7	58	10.4		
HR + /HER-2						
PPBC	4	3.6	7	1.3	**14.711**	** < 0.001**
Parous > 5	17	15.3	266	47.8		
HR /HER-2 +						
PPBC	6	5.4	6	1.1	3.455	0.063
Parous > 5	41	36.9	121	21.8		
TNBC						
PPBC	4	3.6	7	1.3	**1.058**	**0.304**
Parous > 5	25	22.6	86	15.4		

Bold values indicate that they are statistically significant at *P* ≤ 0.05

**Table 7:** Subgroup analysis of the PPBC group versus pCR

Patients characteristic	PPBC	pCR(PPBC vs.Other)	RR	*P* for interaction
	*N*	*n/N*	(95% CI)	
Lymphatic infiltration				
Yes	32	11/32 vs. 74/470	2.183(1.295–3.681)	** < 0.001**
No	8	4/8 vs. 22/157	3.568(1.613–7.893)	
ER				
Positive	17	5/17 vs. 29/349	3.540(1.567–7.994)	** < 0.001**
Negative	23	10/23 vs. 67/278	1.804(1.083–3.006)	
PR				
Positive	16	5/16 vs. 18/273	4.740(2.020–11.122)	** < 0.001**
Negative	24	10/24 vs. 78/354	1.891(1.133–3.156)	
HER-2				
Positive	18	7/18 vs. 42/220	2.037(1.074–3.863)	** < 0.001**
Negative	22	8/22 vs. 54/407	2.741(1.495–5.024)	
KI67				
≤ 15	12	2/12 vs. 25/216	1.440(0.386–5.378)	** < 0.001**
> 15	28	13/28 vs. 71/411	2.688(1.713–4.218)	
Age				
≤ 40	33	14/33 vs. 11/61	2.353(1.208–4.582)	** < 0.001**
> 40	7	1/7 vs. 85/566	0.951(0.153–5.901)	
Stage				
I + II	23	8/15 vs. 73/369	2.696(1.609–4.517)	** < 0.001**
III	17	7/17 vs. 23/258	4.619(2.319–9.201)	

Bold values indicate that they are statistically significant at *P* ≤ 0.05

**Table 8:** Group differences using RECIST as the pathological criteria

RECIST	Postpartum patients				*P*
	PPBC(*n* = 40)		Parous > 5(*n* = 627)		
	*N*	%	*N*	%	
PD	2	5	9	1.4	**0.001**
SD	3	7.5	102	16.2	
PR	20	50.0	420	66.9	
CR	15	35.5	96	15.5	

Bold values indicate that they are statistically significant at *P* ≤ 0.05

### Describe the patient's pCR according to the RECIST standard

**Fig. 8:**
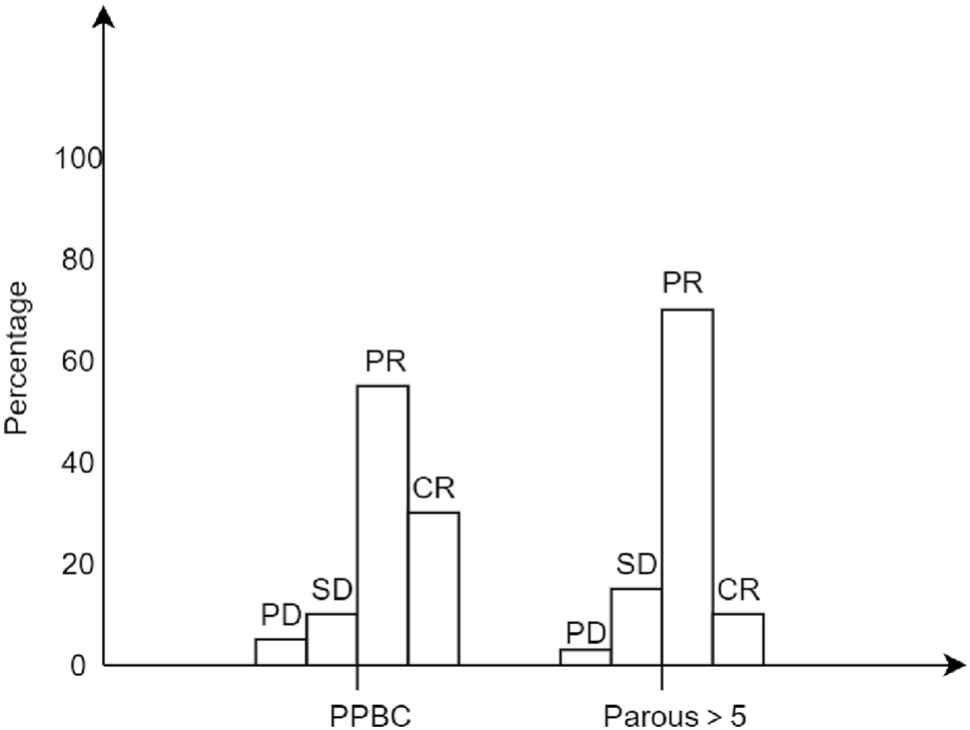
The pathology of the whole group was determined according to RECIST

Clinical efficacy was evaluated according to the efficacy evaluation criteria of the solid tumor criteria (RECIST) version 1.1 (Table 8). Partial remission (PR) and complete remission (CR) were defined as good clinical response; progressive disease (PD) and stable lesions (SD) were defined as poor clinical response (Fig. 8). PR rates were highest in both groups, 50.0% and 66.9%, respectively. The overall outcome of chemotherapy was good, with only 11 patients progressing without improvement after treatment with NAC. Patients with PPBC were twice as likely to achieve CR (35.5% vs. 15.5%), and the difference was statistically significant (*P* < 0.05).

### Effect of a history of abortion and lactation on pCR in BC patients

In the present study, we continued to analyze the effect of other reproductive histories on the pCR of BC patients (Table 9). A total of 437 patients experienced spontaneous or induced abortions. We found that abortion did not affect the response to chemotherapy in BC patients (*P* > 0.05). Only 55 of this group did not breastfeed their infants, and patients were categorized according to the duration of breastfeeding. When BC patients were breastfed for 13–24 months, patients were more likely to achieve pCR, and this correlation was significant (*P* < 0.05). In addition, the sensitivity to chemotherapy was similar in singleton and multiplex patients (*P* > 0.05).

**Table 9:** Effect of other reproductive information on the pCR in BC patients

Patients characteristic	Parous patients				*χ*2	*P*
	pCR (*n* = 111)		NpCR (*n* = 556)			
	*N*	%	*N*	%		
Abortion						
0	39	35.3	191	34.3	0.506	0.918
1	37	24.3	202	36.3		
2	25	31.4	112	20.1		
3	10	9.0	51	9.3		
Lactation						
0	8	7.2	47	8.5	**11.708**	**0.008**
1–12	34	30.6	218	39.2		
13–24	57	51.4	192	34.3		
> 25	12	10.8	99	18.0		
Parity						
1	82	73.9	394	70.9	0.410	0.522
2	29	26.1	162	29.1		

Bold values indicate that they are statistically significant at *P* ≤ 0.05

## Discussion

A total of 714 female patients with BC were selected for this study, of whom approximately 5.6% were patients with PPBC, similar to the epidemiological findings in China. This retrospective study analyzed the response of BC patients to NAC. We found that delivery status was an independent predictor of pCR rates after NAC in BC patients, that PPBC patients were younger at the time of BC diagnosis, that most patients were in an unmenopausal state, had a more significant proportion of the TNBC phenotype, and were more sensitive to chemotherapy than other groups (37.5% vs. 15.3%, *P* < 0.001), these data confirm the importance of the time of the start of the last delivery as a biomarker of NAC outcome in BC patients. When dealing with patients with PPBC, we should strongly recommend chemotherapy to patients and also need to study PPBC as a high-risk subset. By evaluating these factors, physicians can change the treatment decisions of BC patients and achieve appropriate individualized treatment.

Globally, cancer is a growing pregnancy complication, mainly due to the increasing age of women at childbirth (Eastwood-Wilshere et al. 2019). Cancer in women of maternal age remains a major global problem, which is more pronounced in lower socioeconomic countries. Westerners and Asians are different in lifestyle, genetic makeup, and tumor biology, which means that the findings of Western experts may not be apply to Asian populations (Bhoo-Pathy et al. 2013). Thyroid cancer, melanoma, lymphoma, cervical cancer, and BC are the most common PABCs, with only BC and melanoma increasing in recent years, especially PPBC (Johansson et al. 2011).

PPBC remains an under-recognized high-risk BC group, and the age of BC diagnosis is now gradually becoming younger, with a large proportion of patients diagnosed with BC under 45 years of age being PPBC, probably because younger BC patients are more likely to accept becoming mothers after cancer treatment (Colleoni et al. 2002). Goddard found that women had a high risk of having a BC diagnosis within 10 years of delivery and that this effect persisted until 30 years later (Goddard et al. 2019). One study found that PPBC patients had a higher axillary lymph node metastasis rate, and most patients presented with ER-negative, PR-negative, HER-2-positive, or TNBC tumors (Bonnier et al. 1997). This may also be because HR positive patients need endocrine therapy and have a reduced chance of getting pregnant after taking tamoxifen (Petrek et al. 2006). Guinee found that the shorter the interval between pregnancy and diagnosis of BC, the three times more likely to have metastasis and death than other types (Guinee et al. 1994). Some experts believe that the poor prognosis of PPBC patients is due to the young age of the patient and is not related to the presence or absence of pregnancy. Elena believes that this one-size-fits-all approach is incorrect, as pregnancy induces molecular and anatomical changes in the breast that can create a tumor-promoting environment. Experts recommend that the pregnancy history of BC patients must be asked for and fully documented at the time of consultation, and that we need to consider pregnancy as an independent prognostic factor affecting BC. We need to consider pregnancy as an independent poor prognostic factor affecting the survival of patients with BC (Johansson et al. 2011). Some studies have found that pregnancy after diagnosis and treatment of BC is not associated with survival. Therefore it is necessary to investigate PPBC as a risk subset for BC regardless of ER and PR status (Shagisultanova et al. 2022; Matthews and Hamilton 2009; Macias and Hinck 2012).

PPBC is an aggressive subtype characterized by a unique genetic profile, and PPBC affects women far beyond their last pregnancy. There are many potential mechanisms for the poor survival of PPBC patients, most notably the degenerative process of the mammary gland after delivery. Martinson found that changes in the mammary gland after weaning in mice increased tumor growth, invasion, and metastasis persisted for a long time. These changes persisted for a long time (Martinson et al. 2015). The first stage is reversible and occurs 1 to 2 days after weaning, when the lobes of the mammary glands are highly hyperplastic and hypertrophic, the follicular epithelium is arranged in single rows and filled with milk, the fibrous tissue around the milk ducts almost disappears and is replaced by capillaries, the follicles and milk ducts are generally dilated, the epithelial cells of the mammary glands undergo programmed death and regeneration, and redistribution of glands and fat can be observed (Clarkson et al. 2004). The second stage is irreversible and occurs within two weeks after weaning when the extracellular matrix remodeling system allows other cells to continue to die. The mammary gland structure undergoes rapid degenerative changes that are so rapid and extensive that the mammary gland recovers to a volume smaller than its pre-pregnancy level, differentiating the tissue into fat cells (O’Brien et al. 2010; Jindal et al. 2014). The activation of proteases marks this phase, and apoptosis continues throughout the degenerative process (Green and Lund 2005). During weaning-induced cell collapse, occult tumor cells are activated to the mammary stroma and then enter the blood system to spread and establish micrometastases in distant organs (Maller et al. 2013). Macrophages also appear to play a role in breast degeneration, and Condeelis found that macrophages promote tumor cell motility after receiving the recruitment of fibrillar collagen (Condeelis and Pollard 2006). Lyons found abundant fibrillar collagen, high cyclooxygenase-2 expression, and an aggressive phenotype in degenerating mammary glands. NSAIDs can reduce fibrillar collagen production, tumor growth and metastasis of tumor cells to distant sites, by inhibiting high cyclooxygenase-2 (Lyons et al. 2011). In addition to this, the activity of matrix metalloprotease-2 (MMP2) increased during mammary degeneration and human mammary epithelial cells, although constitutively inactive on intact laminin-5, acquired a motile phenotype on MMP2 cleaved laminin-5, contributing to the progression of PPBC (Giannelli et al. 1999). Tamburini found that PD-L1 and PD-1 are highly expressed in degenerated mammary glands, and PD-L1 and its receptor PD-1 can inhibit T-cell responses through the activation of pro-apoptotic signals. The promotion of tumor growth triggered by mammary gland degeneration is reversed after applying anti-PD-L1 therapy (Tamburini et al. 2019; Borges et al. 2020). SEMA7A is a glycosphatidylinositol membrane-anchored protein that promotes the attachment and spreading of multiple cell types. Black found that SEMA7A plays an essential role in degenerating mammary glands and that SEMA7A promotes PPBC progression by regulating tumor-associated COX-2 expression and fibroblast-mediated collagen deposition in the tumor microenvironment (Black et al. 2016). If SEMA7A is knocked down, COX-2 and collagen deposition are also reduced, suggesting that SEMA7A and COX-2 could be new targets for PPBC treatment. Thus, breast degeneration is the most crucial predictor of PPBC. Some experts found that PPBC also has a distinct pattern of visceral metastasis, where circulating tumor cells released after breast degeneration have an additional metastatic advantage that is not reflected in the brain, lung, or bone but is particularly evident in the liver, where metastasis is absent, probably due to a functional link between the breast and the liver (Black et al. 2016; Tarullo Sarah et al. 2020). Goddard found that rodents doubled their liver volume and increased anabolism during post-gestational lactation (Goddard et al. 2017).

It has also been shown that most PPBC is in an intermediate to advanced stage due to a delay in diagnosis (Ishida et al. 1992). Moreira believes that these patients are too young and that the difficulty in diagnosis is due to the different breast tissue itself (Moreira et al. 2010). Pregnancy and breastfeeding can lead to increased in breast volume with palpable masses, and the increased firmness and density can pose a significant challenge on physical examination and imaging (Taylor et al. 2011; Ruiz et al. 2017). During pregnancy, the patient carries a fetus with its own physical needs and risk factors, and the pregnant woman herself can feel anxious and desperate (Ferrari et al. 2018). After the pregnancy, the patient faces the stress of changing identity and must balance caring for the newborn with managing her health and receiving cancer care (Proussaloglou et al. 2023; Loibl et al. 2015). This trade-off can cause women to neglect their health. Lack of common sense by women and physicians is also thought to play an important role in delaying diagnosis. Conditions such as lactational adenomas, malformations, fibroadenomas, and cellulitis often occur during pregnancy and lactation, and these lesions can also influence the imaging physician's judgment (Baker et al. 2001; Presazzi et al. 2015; Lee and Bae 2020). Therefore, when dealing with PPBC patients, patients and doctors should take it seriously.

However, some experts have suggested otherwise, and Amant found that patients diagnosed with BC during pregnancy do not have an increased risk of metastasis and death. Patients with PABC have similar OS to non-pregnant patients (Amant et al. 2013). Litton also found higher OS rates when PPBC patients received appropriate local and systemic therapy (77% vs. 71%), and the authors primarily emphasized the importance of chemotherapy (Litton et al. 2013). In addition, proper exercise strengthens muscles, reduces inflammation and, lowers the risk of disease. The World Health Organization recommends 150 min of moderate-intensity physical activity per week for pregnant and postpartum women to help further heal and improve women's mental health (Bull et al. 2020; Neil-Sztramko et al. 2014). Recent studies have also identified potential targets for treating PPBC, including pathways related to lymphangiogenesis and immunomodulation, offering hope for treating these patients (Lefrère et al. 2021).

In this study, we continued to analyze the effect of breastfeeding and miscarriage on pCR in BC patients. Miscarriage does not affect the sensitivity of chemotherapy in BC patients. In 1980, experts concluded that there was a close association between miscarriage and BC, with undifferentiated mammary cells and fully differentiated cells in menstruating women, and that women who experienced miscarriage were likely to be partially differentiated and more susceptible to carcinogens (Braüner et al. 2013). The American College of Obstetricians and Gynecologists study found that abortion does not increase the risk of having BC. We have found a significant increase in the effectiveness of chemotherapy when BC patients are adequately breastfed. Very few women with BC have breastfed, in large part due to the mother's fear of transmitting cancer cells to her baby through her breast milk; there is no evidence of such transmission, but the average duration of breastfeeding is much shorter than for women without BC due to the psychological stress of having cancer (Breast cancer and breastfeeding 2002). One study found that breastfeeding has a significant protective effect on TNBC with BRCA1 mutation and that breastfeeding significantly reduces the risk of recurrence in BC patients, especially in postmenopausal women (Palmer et al. 2014). Therefore, experts suggest that the duration of breastfeeding should be extended appropriately. In addition, some studies have found that the duration of breastfeeding is shorter in countries with high average socioeconomic status, which is likely to be the reason for the increased incidence of BC in postmenopausal women in developed countries (Victora Cesar et al. 2016). Bernstein found that the endocrinology of a woman's second pregnancy differs from that of her first and that an increase in the number of pregnancies increases the risk of metastasis (Bernstein et al. 1986). In contrast, Woods suggested that an increase in the number of pregnancies reduces the menstrual cycle and maintains the organism in a low estrogenic state, acting as a breast conserving woman like pregnancy and exclusive breastfeeding (Woods et al. 1980).

Our study still has many limitations. First, this is a single-center study with inevitable selection bias, which may not fully represent the entire Chinese population, and subtle differences may not be detected due to the limited number of cases.

Secondly, there is no survival analysis in this study. The prognosis of patients is still the most critical endpoint of the study. In the subsequent study, we need continuous postoperative follow-up. Nevertheless, this study is also a rare study of the NAC response to pregnancy in BC patients in Asian countries. It allows us to better focus on this category of patients and make treatment choices, but we also need further research to confirm our findings and explore the biological background behind it.

## Conclusion

In conclusion, different pregnancy statuses were independent predictors of achieving pCR after BC patients received NAC, and PPBC patients were likelier to achieve pCR than other patients. In previous studies, PPBC was often classified as PABC, hiding its biological characteristics. As a specific group, doctors need to analyze the biological factors of PPBC in more detail, and governments should develop additional policies to help patients achieve precise individualized treatment.

## Data Availability

The datasets generated during and/or analysed during the current study are available from the corresponding author on reasonable request.

## Abbreviations

ALND: Axillary lymph node dissection
BC: Breast cancer
BCS: Breast conserving surgery
BCP: Breast cancer in pregnancy
BMI: Body mass index
DCIS: Ductal carcinoma in situ
ER: Estrogen receptor
HER-2: Human epidermal growth factor receptor-2
HR: Hormone receptor
IDC: Invasive ductal carcinoma
IHC: Immunohistochemical
ILC: Invasive lobular carcinoma
MMP-2: Matrix metalloprotease-2
NAC: Neoadjuvant chemotherapy
PABC: Pregnancy associated breast cancer
PR: Progesterone receptor
pCR: Pathologic complete response
PPBC: Postpartum breast cancer
PSM: Propensity score matching
RECIST: Response Evaluation Criteria In Solid Tumors
ROC: Receiver operating characteristic
SLNB: Sentinel lymph node biopsy
TNBC: Triple negative breast cancer

## Availability of data and materials

The data and materials for this study are authentic and available. All the data can be found in the digital integrated management system of Harbin Medical University Cancer Hospital. Publicly archived datasets analysed or generated during the study may be provided by the corresponding author upon reasonable request.
